# Supplementary material for: Improving Access to Radiotherapy in Gauteng: A Framework for Equitable Cancer Care
Source: Int J Environ Res Public Health. 2025 Jul 3;22(7):1071. doi: 10.3390/ijerph22071071 (PMC12294535; doi:10.3390/ijerph22071071)
Supplement: Supplementary file 1 [file ijerph-22-01071-s001.zip › ijerph-3657801-supplementary.pdf]

Table S1. Participant demographic information.

Participant ID	Age	Gender	Cancer Diagnosis	Education Level	Employment	Distance to Facility[1] (km)
CMJAH PT 1	35	Female	Head & Neck	Degree	Teacher	37
CMJAH PT 2	56	Female	Breast Cancer	Degree	Unemployed	51
CMJAH PT 3	46	Female	Cervical Cancer	Some High School	Driver	25
CMJAH PT 4	57	Female	Head & Neck	Matriculated	Unemployed	32
CMJAH PT 5	55	Female	Cervical Cancer	Some High School	Domestic Worker	19
CMJAH PT 6	68	Female	Breast Cancer	Matriculated	Pensioner	14.2
CMJAH PT 7	43	Female	Cervical Cancer	Some High School	Domestic worker	14
CMJAH PT 8	50	Female	Cervical Cancer	Some High School	Cleaner	45
CMJAH PT 9	72	Female	Cervical Cancer	Some High School	Pensioner	45
CMJAH PT 10	51	Male	Head & Neck	Degree	Teacher	17.4
CMJAH PT 11	69	Male	Prostate Cancer	Matriculated	Pensioner	55
CMJAH PT 12	45	Female	Cervical Cancer	Some High School	General worker	16.4
CMJAH PT 13	53	Female	Cervical Cancer	Some High School	Domestic worker	4
SABH PT 1	54	Female	Cervical Cancer	Some High School	Self-employed (selling clothes)	16
SABH PT 2	75	Female	Cervical Cancer	Primary school	Pensioner	46
SABH PT 3	54	Female	Cervical Cancer	Some High School	Domestic worker	65
SABH PT 4	19	Male	Head & Neck	Some High School	Unemployed	55
SABH PT 5	43	Female	Cervical Cancer	Matriculated	General work (Road Maintenance)	16
SABH PT 6	26	Female	Cervical Cancer	Some High School	Unemployed	41
SABH PT 7	46	Female	Cervical Cancer	Diploma	Clerk	41
SABH PT 8	68	Male	Prostate Cancer	Matriculated	Pensioner	39
SABH PT 9	33	Female	Cervical Cancer	Matriculated	Retail	41
SABH PT 10	58	Female	Cervical Cancer	Primary school	Self-employed (selling vegetable)	114
SABH PT 11	42	Female	Breast Cancer	Degree	Teacher	42
SABH PT 12	50	Female	Cervical Cancer	Diploma	Lab Assistant	41

Participant ID: A unique identifier for each participant, **Age**: The participant's age at the time of the study, **Gender**: The participant's gender; **Cancer Diagnosis**: The type of cancer the participant has been diagnosed with; **Education Level**: The highest level of education the participant has completed; **Distance to Facility (km)**: The approximate distance in kilometers between the participant's residence and the radiotherapy facility [1] Distance was calculated using Google Maps from the participant's reported address to the main entrance of the radiotherapy facility.
